# Extension of cervical screening intervals with primary human papillomavirus testing: observational study of English screening pilot data

**DOI:** 10.1136/bmj-2021-068776

**Published:** 2022-05-31

**Authors:** Matejka Rebolj, Kate Cuschieri, Christopher S Mathews, Francesca Pesola, Karin Denton, Henry Kitchener, HPV pilot steering group, Tracey-Louise Appleyard, Margaret Cruickshank, Kay Ellis, Chris Evans, Viki Frew, Thomas Giles, Alastair Gray, Miles Holbrook, Katherine Hunt, Tanya Levine, Emily McBride, David Mesher, Timothy Palmer, Janet Parker, Elizabeth Rimmer, Hazel Rudge Pickard, Alexandra Sargent, David Smith, John Smith, Kate Soldan, Ruth Stubbs, John Tidy, Xenia Tyler, Jo Waller

**Affiliations:** 1 Cancer Prevention Group, School of Cancer and Pharmaceutical Sciences, Faculty of Life Sciences and Medicine, King’s College London, London, UK; 2 Scottish HPV Reference Laboratory, Royal Infirmary of Edinburgh, NHS Lothian Scotland, Edinburgh, UK; 3 Severn Pathology, Southmead Hospital, North Bristol NHS Trust, Bristol, UK; 4 Division of Cancer Sciences, University of Manchester, Manchester, UK

## Abstract

**Objectives:**

To provide updated evidence about the risk of cervical intraepithelial neoplasia grade 3 or higher (CIN3+) and cervical cancer after a negative human papillomavirus (HPV) test in primary cervical screening, by age group and test assay.

**Design:**

Observational study.

**Setting:**

Real world data from the English HPV screening pilot’s first and second rounds (2013-16, follow-up to end of 2019).

**Participants:**

1 341 584 women.

**Interventions:**

Cervical screening with HPV testing or liquid based cytological testing (cytology or smear tests). Women screened with cytology were referred to colposcopy after high grade cytological abnormalities or after borderline or low grade abnormalities combined with a positive HPV triage test. Women screened with HPV testing who were positive were referred at baseline if their cytology triage test showed at least borderline abnormalities or after a retest (early recall) at 12 and 24 months if they had persistent abnormalities.

**Main outcome measures:**

Detection of CIN3+ and cervical cancer after a negative HPV test.

**Results:**

For women younger than 50 years, second round detection of CIN3+ in this study was significantly lower after a negative HPV screen in the first round than after cytology testing (1.21/1000 *v* 4.52/1000 women screened, adjusted odds ratio 0.26, 95% confidence interval 0.23 to 0.30), as was the risk of interval cervical cancer (1.31/100 000 *v* 2.90/100 000 woman years, adjusted hazard ratio 0.44, 0.23 to 0.84). Risk of an incident CIN3+ detected at the second screening round in the pilot five years after a negative HPV test was even lower in women older than 50 years, than in three years in women younger than 50 years (0.57/1000 *v* 1.21/1000 women screened, adjusted odds ratio 0.46, 0.27 to 0.79). Women with negative HPV tests at early recall after a positive HPV screening test without cytological abnormalities had a higher detection rate of CIN3+ at the second routine recall than women who initially tested HPV negative (5.39/1000 *v* 1.21/1000 women screened, adjusted odds ratio 3.27, 95% confidence interval 2.21 to 4.84). Detection after a negative result on a clinically validated APTIMA mRNA HPV test was similar to that after clinically validated cobas and RealTime DNA tests (for CIN3+ at the second round 1.32/1000 *v* 1.14/1000 women screened, adjusted odds ratio 1.05, 0.73 to 1.50).

**Conclusions:**

These data support an extension of the screening intervals, regardless of the test assay used: to five years after a negative HPV test in women aged 25-49 years, and even longer for women aged 50 years and older. The screening interval for HPV positive women who have negative HPV tests at early recall should be kept at three years.

## Introduction

Randomised trials have provided key evidence indicating that tests for human papillomavirus (HPV) have an increased sensitivity over cytological tests (cytology or smear tests) in cervical screening. A meta-analysis of these longitudinal trials showed that by increasing the detection of high grade cervical intraepithelial neoplasia (CIN grade 2 or higher) at an earlier stage, HPV testing can prevent more cases of cervical cancer and therefore, screening intervals can be extended.^1^ This observation was aggregated across all screening ages and tests combined, given the limitations imposed by the small number of women included in the randomised trials: about 94 000 screened with HPV testing and about 82 000 screened with cytology.

Since then, questions remain as to whether screening intervals after a negative HPV test should vary with a woman’s age^2^ or with the type of test assay.^3^ The urgency to find answers to these questions is emphasised by the fact that some screening programmes (such as programmes in England, Scotland, and Wales) have announced plans to extend the intervals for some age groups, and that most HPV based screening programmes internationally do not use Hybrid Capture 2 or GP5+/6+ polymerase chain reaction tests, which were the two HPV tests initially validated in the trials.

The English Cervical Screening Programme rolled out HPV testing as the primary screening method in December 2019, after a successful pilot study.^4^ Using data from the early phase of that pilot, we reported on the reduction of CIN grade 3 or higher (CIN3+) and cervical cancer detection after a negative HPV test in women younger than 50 years.^5^ Since then, the data available for study have more than doubled in size to include about 1.3 million women and have been linked with the national cancer registry. These data have allowed for a detailed breakdown of the findings, which provides an evidence based approach to extend the intervals between screening tests for women with negative HPV results as compared with women with negative cytology results. We assessed the detection of CIN3+ and cervical cancer after two rounds of screening under routine conditions with either HPV testing or liquid based cytology depending on the women’s age and whether the HPV test assay was DNA or mRNA based.

## Methods

### Background of the English HPV pilot 

The English Cervical Screening Programme routinely invites women aged 25-49 years every three years and women aged 50-64 years every five years for cervical screening. All samples are taken by a trained healthcare professional. The pilot, which has been described in detail elsewhere,^5^ ^6^ ^7^ did not change these conditions. Briefly, six screening laboratories partially converted their primary screening activity from cytology to HPV testing. For operational reasons, the participating laboratories selected general practices for this conversion. The cytology and HPV testing platforms used by each laboratory are shown in table 1.

**Table 1:** Numbers of women screened at age 24-64 years in the English HPV screening pilot’s first round, by laboratory site and screening test

Laboratory	Cytology system	No of women screened with cytology	HPV test	No of women screened with HPV testing
A	ThinPrep	117 643	APTIMA, Hybrid Capture 2	50 923*
B	SurePath	85 698	APTIMA	41 027
C	ThinPrep	154 547	RealTime	46 764
D	SurePath	294 451	RealTime, cobas	143 263^†^
E	ThinPrep	128 941	cobas	32 108
F	SurePath	156 421	cobas	89 798

HPV=human papillomavirus.

* The laboratory used either APTIMA or Hybrid Capture 2 for primary screening before the end of 2015. Information on which HPV test was used for each sample during that period was not available. The number of women screened with APTIMA from January to December 2016 was 14 167.

† The laboratory used the RealTime test until March 2016 and the cobas test from April 2016 onward. The number of women screened with HPV testing from April to December 2016 was 22 936.

Women who were HPV positive after screening with HPV testing were referred to colposcopy at baseline if their cytology triage test showed at least borderline abnormalities in squamous or glandular cells, or after a retest (that is, early recall) 12 months after the primary screening if they remained HPV positive and developed at least borderline abnormal cytological results. In three of the six laboratories if, despite negative cytology results, women were still positive for HPV types 16 or 18 or both at 12 months, women were referred for colposcopy.^7^ Otherwise, if still positive for HPV at 24 months, they were referred for colposcopy regardless of their cytological results. Women screened with cytology were referred to colposcopy after high grade cytological abnormalities or after borderline or low grade abnormalities combined with a positive HPV triage test. Neither of the screening protocols in this real life setting allowed for colposcopies in women with negative screening tests, unless they presented with symptoms suggestive of cervical cancer.

Women were followed up across two consecutive screening rounds. The first round took place between May 2013 and December 2016 and was followed by the second round (after a time interval that was dependent on the woman’s age). Women aged 24-49 years were invited for a new screening round in three years after a negative screen, women aged 50-59 years were invited for a new screening round in five years, and women aged 60-64 years were not invited for the second screening round because programme eligibility ended at age 65 years. Any follow-up and new primary screening tests for women aged 24-64 years, together with the associated cytological and histological diagnoses, were monitored until the end of December 2019. Supplementary figure S1 shows the completeness of data for early recall in the first round and for primary screening and early recall in the second round by age group.

### Data retrieval

Data for screening tests and histological outcomes until the end of 2019 were retrieved from the laboratory information systems. Data for cervical cancers (ICD-10 (international classification of diseases, 10th revision) code C53) and CIN3 (ICD-10 code D06) diagnosed between 1995 and 2018 were retrieved from the English National Cancer Registration and Analysis Service.^8^ We linked data belonging to the same woman by their unique English NHS numbers. Throughout this analysis, information on CIN diagnoses made during the pilot was based on the laboratory data while the source of data for cervical cancers was from the English National Cancer Registration and Analysis Service.

### Participant exclusion criteria

The reason for taking the sample was not available in the retrieved data. To identify primary screening samples and remove samples that had followed a recent abnormality, we excluded women with cervical cancer preceding their first pilot sample, as well as women with CIN3 reported to the English National Cancer Registration and Analysis Service in the preceding three years. Additionally, women were excluded if they had cytology or HPV test results registered in the laboratory data in the preceding two years, or had additional information that indicated that the first pilot sample was taken for follow-up of a recent abnormality.

### Statistical analysis

We studied data for women aged 24-49, 50-59, and 60-64 years. Age was determined at the time the screening sample was taken in the first round. Women aged 24 years were included because the cervical screening programme sends the first invitation six months before their 25th birthday.

We determined the outcomes of screening across two consecutive rounds and compared continued screening with HPV testing with continued screening with cytology. In the first round, women who had positive screening tests and were subsequently triaged to colposcopy at baseline or at one of the early recalls could receive a diagnosis of CIN3 or cervical cancer detected by screening. A higher detection at this stage should translate into a lower detection after negative screening tests. Women with negative screening tests could receive diagnoses of interval cervical cancer, usually following symptoms, any time before the routine recall for a new primary screening test (the second screening round in the pilot) in three or five years, depending on their age. Women who were not diagnosed with cervical cancer in the interval between screening were eligible for the invitation to the second screening round. A subset among these women could have become infected with HPV or developed cytological abnormalities that would be detectable at the second round. Women with abnormalities detected at the second round were triaged to colposcopy and could receive a second round diagnosis of screen detected CIN3 or cervical cancer. Owing to limited data availability, women who were referred to routine recall after colposcopy in the first round and those who did not attend recommended early recall tests were not included in the analysis of the second round data.

For both cytology and HPV testing, we determined the detection of (squamous or glandular) CIN3+ and cervical cancer after a positive screening test in each round (first *v* second). Second rounds were included for all women with negative screening tests in the first round. A proportion of women screened with cytology in the first round were switched to HPV testing in the second round as part of a national strategy implemented from 2018 onwards, to mitigate a shortage of cytoscreeners.^9^ ^10^ To estimate second round detection of CIN3+ and cancer associated with continuous cytology versus HPV screening, we included second round data only if women had been screened with the same test in both rounds. We report the characteristics of women attending the second round and those who were screened with the same test as in the first round in supplementary tables S2 and S3. For women screened with HPV testing, we also determined the detection of CIN3+ and cervical cancer by HPV test assay, and for the mRNA viral target (APTIMA) versus the DNA viral target (cobas or RealTime combined). Cobas and RealTime assays detect the DNA of HPV 16 and HPV 18 separately and the DNA of the other 12 high risk genotypes (31, 33, 35, 39, 45, 51, 52, 56, 58, 59, 66, 68) in combination. APTIMA reports the detection of mRNA from the same 14 HPV genotypes in combination. For this analysis, we excluded women screened before January 2016 in laboratory site A because of insufficient information on the allocation of APTIMA and Hybrid Capture 2 tests. We also determined the detection of CIN3+ at the second round for women whose first round screening samples were HPV positive but cytologically negative, and were returned to routine recall after they had tested negative for HPV at the 12 or 24 month early recall.

We studied the relative detection of CIN3+ and cancer using odds ratios with associated 95% confidence intervals from logistic regression. If the number of cases was sufficient (15 per explanatory variable), these odds ratios were adjusted for women’s ages, laboratory site as a proxy for unmeasured local characteristics associated with screening outcomes, and decile of the Index of Multiple Deprivation, which is a standard indicator of socioeconomic deprivation based on postcodes in England.^11^ For comparison with HPV tests, the odds ratios were adjusted for cytology brand, women’s age, and Index of Multiple Deprivation decile. We excluded from the analysis a small number of women whose information on Index of Multiple Deprivation was missing (<1%).

In women with a negative screening test in the first round, we reported the frequency of cervical cancer diagnosed any time before the second round (referred to here as an interval cancer), per 100 000 woman years at risk. Woman years were counted from the date of the negative screening test until the date of cancer diagnosis, date of a screening test in the second round for ages 24-59 years, date of colposcopy for ages 60-64 years (if any at all), or 31 December 2018 (that is, end of the English National Cancer Registration and Analysis Service data), whichever came first. The relative risk was estimated by means of a hazard ratio using Cox regression and adjusted as previously mentioned. The assumption of proportional hazards was tested with Schoenfeld residuals for each covariate as well as globally and was found to be satisfied for all reported models.

To help develop policy recommendations, we assumed that the detection of CIN3+ and cervical cancer observed after negative cytology represents the risk that is acceptable by the healthcare service. We also assumed that women who have negative HPV test results but have similar risks as women with negative cytology of CIN3+ or cervical cancer, or both, should be rescreened with the same routine recall intervals, whereas for women with lower observed risks, extension of the routine recall intervals is possible. We used R version 3.6.1 for all analyses.

### Patient and public involvement

Neither patients nor the public were involved in the management of this study. The safety and acceptability of the screening and early recall protocols had been shown in the ARTISTIC trial,^12^ whereas the aim of the pilot study was to demonstrate the feasibility of HPV based screening in a real life setting and on a large scale. Nevertheless, the pilot study included a comprehensive substudy of psychosexual responses to HPV based screening, which has reported its findings separately.^13^ ^14^ ^15^ ^16^ ^17^

## Results

### First screening round, for women aged 24-59 years

Of 1 341 584 women, the first round included 300 677 aged 24-49 years screened with HPV testing and 706 820 screened with cytology (table 2; supplementary table S1 and fig S2). HPV testing detected 5313 women with CIN3+, while cytology detected CIN3+ in 8232 women (17.67 *v* 11.65/1000 women screened; adjusted odds ratio 1.55, 95% confidence interval 1.50 to 1.61). Cervical cancer was detected in 259 women by HPV testing and 441 women by cytological testing (0.86 *v* 0.62/1000 screened; 1.38, 1.18 to 1.61).

**Table 2:** Test performance among women screened at age 24-59 years in the English HPV screening pilot’s first round; detection of CIN3+ and cervical cancer after a positive screening test by screening round, screening test, and age group. Includes first rounds in the pilot in 2013-16, and second rounds in the pilot by end of 2019 for women with negative screening test outcomes in the first round (information on cervical cancer available until the end of 2018)

After a positive screen	Age 24-49 years				Age 50-59 years		
	HPV testing (No per 1000 screened)	Cytology (No per 1000 screened)	HPV testing *v* cytology (OR_adj_ (95% CI))*		HPV testing (No per 1000 screened)	Cytology (No per 1000 screened)	HPV testing *v* cytology (OR_adj_ (95% CI))*
**First screening round**							
No of women screened	300 677	706 820	—		79 040	175 973	—
No of CIN3+ lesions detected	5313 (17.67)	8232 (11.65)	1.55 (1.50 to 1.61)		219 (2.77)	332 (1.89)	1.56 (1.31 to 1.85)
No of cervical cancers detected	259 (0.86)	441 (0.62)	1.38 (1.18 to 1.61)		31 (0.39)	52 (0.30)	1.41 (0.90 to 2.21)
**Second screening round** **^†^**							
No of women screened	188 318	260 266	—		24 550	22 195	—
No of CIN3+ lesions detected	227 (1.21)	1177 (4.52)	0.26 (0.23 to 0.30)		14 (0.57)	20 (0.90)	0.63 (0.32 to 1.25)^‡^
No of cervical cancers detected	1 (0.01)	54 (0.21)	0.02 (0.00 to 0.17)		1 (0.04)	2 (0.09)	0.45 (0.04 to 4.98)^‡^

CI=confidence interval; CIN=cervical intraepithelial neoplasia; HPV=human papillomavirus; OR_adj_=adjusted odds ratio.

* Odds ratios (for the detection of CIN3+ or screen detected cancer after a positive screen) were adjusted for age in years, decile of the Index of Multiple Deprivation, and laboratory site. Unadjusted estimates were similar as adjusted estimates and are reported in supplementary table S4.

† Women were selected into the analysis of the pilot’s second round if they had a negative screening test in the first round and were screened with the same test, cytology or HPV, in both rounds. Because of the national mitigation strategy from 2018 onward, more women initially screened with cytology in the pilot’s first round were assigned to HPV testing in the second round than the was the case the other way around.

‡ Unadjusted estimates owing to a small number of cases.

**Fig 1:**
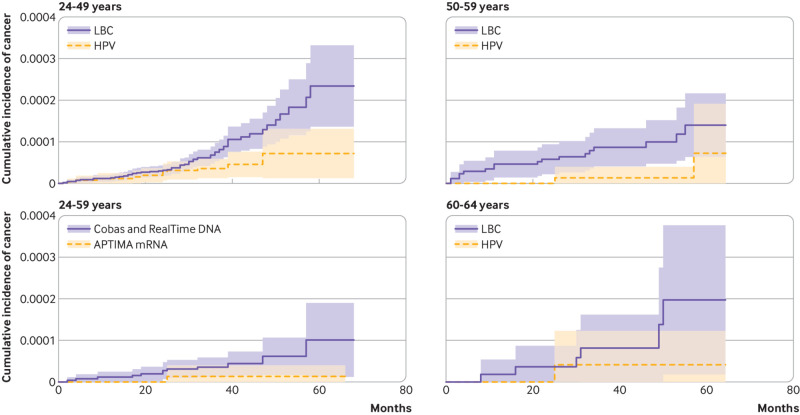
Cumulative incidence of interval cervical cancer after a negative screening test result in the first round, by age group or by HPV test type. Interval cancers are those diagnosed between the first and second screening rounds. Shaded areas denote 95% confidence intervals. LBC=liquid-based cytology; HPV=human papillomavirus

Eleven interval cancers were diagnosed in 837 960 woman years after negative HPV tests taken at age 24-49 years, and 62 in 2 135 315 woman years after negative cytology (1.31 *v* 2.90/100 000 woman years; adjusted hazard ratio 0.44, 95% confidence interval 0.23 to 0.84; table 3, fig 1). At the second screening round, the detection of CIN3+ was lower for women who had screened negative with HPV testing in the first round (227 of 188 318) than in women who had screened negative with cytology (1177 of 260 266; 1.21/1000 screened *v* 4.52/1000; adjusted odds ratio 0.26, 95% confidence interval 0.23 to 0.30; table 2). The same was the case for the detection of cervical cancer, one woman versus 54 women (0.01/1000 screened *v* 0.21/1000 screened; 0.02, 0.00 to 0.17).

**Table 3:** Interval cervical cancer diagnosed between the first and second screening rounds after a negative screen*, by screening test and age group. Includes women with a negative screening test in the English HPV screening pilot’s first round in 2013-16, with information on cervical cancer available until the end of 2018

Age group (years)*	HPV testing	Cytology	HPV testing *v* cytology (adjusted hazard ratio (95% CI)†
**24-49**			
Total No of woman years	837 960	2 135 315	—
Total No of cervical cancers diagnosed (No of cervical cancers diagnosed per 100 000 woman years)	11 (1.31)	62 (2.90)	0.44 (0.23 to 0.84)
**50-59**			
Total No of woman years	281 369	631 584	—
Total No of cervical cancers diagnosed (No of cervical cancers diagnosed per 100 000 woman years)	2 (0.71)	17 (2.69)	0.26 (0.06 to 1.15)‡
**60-64**			
Total No of woman years	86 712	194 688	—
Total No of cervical cancers diagnosed (No of cervical cancers diagnosed per 100 000 woman years)	1 (1.15)	6 (3.08)	0.39 (0.05 to 3.20)‡

CI=confidence interval; HPV=human papillomavirus.

* Average numbers of woman years at risk at age 24-49 year were 3.3 years for HPV testing and 3.2 years for cytology; corresponding numbers were 3.8 years and 3.7 years, respectively, at age 50-59 years; and 3.8 years and 3.6 years, respectively, at age 60-64 years.

† Hazard ratios (for interval cancer diagnosis between first and second screening rounds after a negative screen) were adjusted for age in years, decile of the Index of Multiple Deprivation, and laboratory site. Unadjusted estimates were similar as adjusted estimates and are reported in supplementary table S4.

‡ Unadjusted estimates owing to a small number of cases.

In total, 45% (12 709) of women aged 24-49 years with HPV positive and cytologically negative screening samples had a negative HPV screening test either at the 12 month early recall (10 248, 81%) or 24 month early recall (2461, 19%; not tabulated). Among these 12 709 women, 5380 had been screened in the second round, with the detection of 29 CIN3+ lesions (5.39/1000 screened), none of which was cancer. Compared with 188 318 women who had a negative HPV screening test in the first round (table 2), the second round risk was higher for women with negative HPV tests at early recall (adjusted odds ratio 3.27, 95% confidence interval 2.21 to 4.84). Of the 5380 women with the second round data, 4181 were screened in one of the four laboratories that used HPV genotyping tests. Here, CIN3+ was detected at the second round in 8.9/1000 women with HPV16/18 infections, whereas this proportion was 4.5/1000 in women infected with other 12 high risk genotypes. Although the risk per 1000 women was higher for HPV16/18 infections, about two thirds of all CIN3+ lesions were detected in women infected with other genotypes (7 *v* 15, respectively).

At age 50-59 years, 79 040 women were screened with HPV testing and 175 973 with cytology in the pilot’s first round (table 2); CIN3+ was detected in 219 and 332 women, respectively (2.77 *v* 1.89/1000 screened; adjusted odds ratio 1.56, 95% confidence interval 1.31 to 1.85). With 31 and 52 cases detected in women screened with HPV testing and those screening with cytology, respectively (0.39 *v* 0.30/1000 screened), the adjusted odds ratio for the detection of cervical cancer in the first round was 1.41 (95% confidence interval 0.90 to 2.21). Two women had interval cancer diagnosed in the subsequent 281 369 woman years after a negative HPV test, as did 17 women in 631 584 woman years after negative cytology (0.71 *v* 2.69/100 000 woman years; hazard ratio 0.26, 95% confidence interval 0.06 to 1.15; fig 1). 

In the second round, CIN3+ was detected in 14 of 24 550 women screened with HPV testing and in 20 of 22 195 women screened with cytology (0.57 *v* 0.90/1000 screened; odds ratio 0.63, 95% confidence interval 0.32 to 1.25). In the second round, one cancer was detected with HPV screening and two were detected with cytology. Of 1570 women who were HPV positive at screening but HPV negative at early recall, 98 had had a second round screen by the end of 2019, with no reported CIN3+ cases (not tabulated).

With HPV testing in the first round, the detection of both CIN3+ (adjusted odds ratio 0.16, 95% confidence interval 0.14 to 0.18) and cervical cancer (0.46, 0.32 to 0.67; based on the data reporting the detection of lesions in table 2) was significantly lower at age 50-59 years than at age 24-49 years. Significantly fewer cases of CIN3+ were also detected at the second round screen five years after negative HPV screening at age 50-59 years than at the second round three years after negative HPV screening at age 24-49 years (adjusted odds ratio 0.46, 95% confidence interval 0.27 to 0.79).

We compared 51 639 women screened with an HPV mRNA test (APTIMA) and 292 831 women screened with an HPV DNA test in the first round (cobas or RealTime; table 4). Detection was similar for the two types of tests for CIN3+ (adjusted odds ratio 1.04, 95% confidence interval 0.96 to 1.12) and cancer (0.95, 0.67 to 1.34) in the first round, as well as for detection of CIN3+ in the second round after negative tests in the first round (1.05, 0.73 to 1.50). One woman had interval cancer diagnosed after a negative APTIMA test versus 12 women after negative cobas or RealTime tests, resulting in a hazard ratio of 0.51 (95% confidence interval 0.07 to 3.91; table 5, fig 1). The adjusted odds ratio for second round detection of CIN3+ was 1.38 (95% confidence interval 0.91 to 2.08) for APTIMA and 1.65 (1.22 to 2.23) for RealTime compared with cobas. For the incidence of interval cancer, the hazard ratio were 0.35 (95% confidence interval 0.04 to 2.84) for APTIMA and 0.40 (0.12 to 1.33) for RealTime compared with cobas.

**Table 4:** Test performance among women screened at age 24-59 years in the English HPV screening pilot’s first round; detection of CIN3+ and cervical cancer after a positive screening test, by type of HPV detection target and screening round. Includes first rounds in the pilot in 2013-16, and second rounds in the pilot by end of 2019 for women with negative screening test outcomes in the first round (information on cervical cancer available until the end of 2018)

Screening round	APTIMA (No per 1000 screened)*	RealTime (No per 1000 screened)*	Cobas (No per 1000 screened)*	RealTime or cobas combined (No per 1000 screened)*	APTIMA *v* cobas (OR_adj_ (95% CI)) †	RealTime *v* cobas (OR_adj_ (95% CI)) †	APTIMA *v* RealTime (OR_adj_ (95% CI)) †	APTIMA *v* RealTime or cobas combined (OR_adj_ (95% CI)) †
**First screening round**								
No of women screened	51 639	157 090	135 741	292 831	—	—	—	—
No of CIN3+ lesions detected	815 (15.78)	2155 (13.72)	2026 (14.93)	4181 (14.28)	1.03 (0.95 to 1.12)	1.06 (0.99 to 1.13)	1.00 (0.92 to 1.09)	1.04 (0.96 to 1.12)
No of cervical cancers detected	37 (0.72)	131 (0.83)	85 (0.63)	216 (0.74)	1.15 (0.78 to 1.70)	1.44 (1.10 to 1.90)	0.80 (0.56 to 1.15)	0.95 (0.67 to 1.34)
**Second screening round**‡								
No of women screened	26 554	93 006	72 187	165 193	—	—	—	—
No of CIN3+ lesions detected	35 (1.32)	123 (1.32)	65 (0.90)	188 (1.14)	1.38 (0.91 to 2.08)	1.65 (1.22 to 2.23)	0.86 (0.59 to 1.25)	1.05 (0.73 to 1.50)
No of cervical cancers detected	0 (0)	1 (0.01)	1 (0.01)	2 (0.01)	—	0.78 (0.05 to 12.41)§	—	—

CI=confidence interval; CIN3+=cervical intraepithelial neoplasia grade 3 or higher; HPV=human papillomavirus; OR_adj_=adjusted odds ratio.

* Data for APTIMA includes all HPV tests from the two laboratories using this test, except for tests made before January 2016 in Laboratory A (table 1). Data for RealTime includes primary HPV tests from Laboratory D until the end of March 2016. Primary HPV tests from Laboratory D from April to end of December 2016 were counted as cobas tests.

† Odds ratios (for the detection of CIN3+ or screen detected cervical cancer after a positive screen) were adjusted for age in years, decile of the Index of Multiple Deprivation, and brand of cytology. Unadjusted estimates were similar as adjusted estimates and are reported in supplementary table S5.

‡ Women were selected in the analysis of the second round if they had a negative HPV test in the first round and were screened with an HPV test also in the second round.

§ Unadjusted estimate owing to a smaller number of cases.

**Table 5:** Interval cancer diagnosed between the first and second screening rounds after a negative screen, by type of HPV detection target. Includes women with a negative screening test in the English HPV screening pilot’s first round in 2013-16, with information on cervical cancer available until the end of 2018

After a negative screen for HPV	APTIMA	RealTime	cobas	RealTime or cobas combined	APTIMA *v* cobas (hazard ratio (95% CI))	RealTime *v* cobas (hazard ratio (95% CI))	APTIMA *v* RealTime (hazard ratio (95% CI))	APTIMA *v* RealTime or cobas combined (hazard ratio (95% CI))
Total No of woman years	143 049	483 578	385 193	868 771	—	—	—	—
Total No of cervical cancers diagnosed (No of cervical cancers diagnosed per 100 000 woman years)	1 (0.70)	4 (0.83)	8 (2.08)	12 (1.38)	0.35 (0.04 to 2.84)*	0.40 (0.12 to 1.33)*	0.73 (0.08 to 6.21)*	0.51 (0.07 to 3.91)*

CI=confidence interval; HPV=high risk human papillomavirus.

* Unadjusted estimate owing to a small number of cases.

### Final screening round, for women aged 60-64 years

HPV testing was used in the final screening round for 24 166 women and cytology for 54 908 (table 6). CIN3+ detection was higher with HPV testing (62 women) than with cytological testing (83; 2.57 *v* 1.51/1000 screened; adjusted odds ratio 1.69, 95% confidence interval 1.21 to 2.37). After a positive screen, cancer was detected in nine women after HPV testing compared with in 23 after cytological testing (0.37 *v* 0.42/1000 screened); odds ratio 0.89, 95% confidence interval 0.41 to 1.92). Among women with a negative screening test, interval cervical cancer was observed in one woman after HPV testing and in six after cytological testing (1.15 *v* 3.08/100 000 woman years; hazard ratio of 0.39, 95% confidence interval 0.05 to 3.20) (table 3). Of 344 women who tested HPV positive at screening but who had negative HPV tests at early recall, none received a diagnosis of cervical cancer by the end of 2018 (not tabulated).

**Table 6:** Test performance among women screened at age 60-64 years in the English HPV screening pilot’s first round; detection of CIN3+ and cervical cancer after a positive screening test, by screening test. Includes first rounds in the pilot in 2013-16, with follow-up for investigation of abnormalities by the end of 2019 (information on cervical cancer available until the end of 2018)

After a positive screen	HPV testing	Cytology	HPV testing *v* cytology (OR_adj_ (95% CI))^*^
No of women screened	24 166	54 908	
No of CIN3+ detected (No of CIN3+ detected per 1000 women screened)	62 (2.57)	83 (1.51)	1.69 (1.21 to 2.37)
No of cervical cancers detected (No of cervical cancers detected per 1000 women screened)	9 (0.37)	23 (0.42)	0.89 (0.41 to 1.92)^†^

CI=confidence interval; CIN3+=cervical intraepithelial neoplasia grade 3 or higher; HPV=high risk human papillomavirus; OR_adj_=adjusted odds ratio.

* Odds ratios (for the detection of CIN3+ or screen detected cervical cancer after a positive screen) were adjusted for age in years, decile of the Index of Multiple Deprivation, and laboratory site. Unadjusted estimates were similar to adjusted estimates and are reported in supplementary table S4.

† Unadjusted estimates owing to a smaller number of cases.

## Discussion

### Principal findings

Using data that represented more than four million woman years at risk after a negative screening test, we estimated the incidence of CIN3+ and cervical cancer in different groups of women undergoing routine cervical screening. We found that at age 24-49 years, incidence is around 74% lower after a negative HPV screen than after a negative cytological result. We also observed that, after a negative HPV screening test, the incidence of CIN3+ at the subsequent screening round was around 50% lower in women aged 50-59 years than in women screened at age 24-49 years. Furthermore, the differences in the detection of CIN3+ and cancer between the different HPV tests were relatively small.

### Strengths and limitations of the study

The pilot’s strength was that the study was undertaken within a real life setting of a national screening programme, using the same commercial HPV tests, and the same screening and diagnostic protocols that are routinely used in the English programme. Furthermore, the study included several times as many women as the randomised trials that formed the evidence base for the widespread adoption of HPV testing.^1^ An early analysis relying solely on laboratory data suggested an increased safe period after a negative HPV test,^5^ which helped the English Cervical Screening Programme to proceed with a national roll-out of HPV testing for all women.^18^ Our analysis provides substantially more data for the period after a negative screening test, with cancer diagnoses, whether detected by screening or by symptoms, confirmed independently by the national cancer registry. 

A relative weakness of the pilot was that test allocation was not randomised. Although our estimates of the relative detection for HPV tests versus cytology were adjusted for the women’s ages, deprivation, and a proxy for screening technology, some residual confounding could have remained. Despite the large size of the study, the observed numbers of women with cancer in specific subgroups at low risk (eg, among older women with negative screening tests) were nevertheless small. Even in those women, the regression models comparing the risks between different screening tests converged but to avoid overfitting, the analyses remained unadjusted for the sociodemographic differences between women undergoing screening. Data from the second round early recall were scarce particularly for women aged 50-59 years. In the second round, however, histology data were available for most baseline colposcopies, which in turn probably represented the majority of CIN3+ lesions detected in the second round. This assumption is supported by more complete, second round data from women aged 24-49 years who were screened early in the pilot, in whom two thirds of the detected CIN3+ lesions were detected at baseline colposcopies.

### Comparison with other studies and policy implications

These updated data^5^ for women younger than 50 years with almost three million woman years of observation confirmed that the incidence of CIN3+ three years after a negative HPV test is about 74% lower than after negative cytology (adjusted odds ratio 0.26).^1^ ^5^ ^19^ ^20^ ^21^ ^22^ They support the English Cervical Screening Programme’s planned extension of the screening interval from three to five years for HPV negative women younger than 50 years and align with practice in other countries. The decision to extend the screening interval to five years was made by the UK National Screening Committee and was informed, primarily, by the early evidence from this pilot study^5^ and the randomised controlled trial ARTISTIC, which was also conducted within the English Cervical Screening Programme. In ARTISTIC, the three year reduction in CIN3+ detection among all women who screened HPV negative at age 20-64 was 66% (0.9 *v* 2.6/1000) compared with all women who screened cytology negative.^12^ At six years, the corresponding comparison of the cumulative rate of CIN3+ was 2.8 versus 6.3/1000 screened, with the cumulative incidence at six years among HPV negative women almost identical to the cumulative rate at three years for women with negative cytology results.

The ARTISTIC trial showed that HPV negative women over age 50 years have a low risk of CIN3+ at the second round, with around 0.5/1000 screened. By the third consecutive round in six years, this risk remained stable.^23^ In the pilot study, almost 25 000 women aged 50-59 years who were negative for HPV attended the second round in five years, and their CIN3+ detection was similar to that in the ARTISTIC trial (0.6/1000 screened). This detection is about half of that observed in younger women and is consistent with the findings of a US study that included women who had been through one or more rounds of screening with HPV testing.^22^ Although CIN lesions are more difficult to detect in postmenopausal women than in younger women, the risk of cancer at older ages remains low for up to 25 years after a negative screen.^24^ Additional data from the pilot for women who underwent their second screening round after 2019 would provide further reassurance about the robustness of this conclusion. However, the currently available data and other studies such as the ARTISTIC trial^12^ point to the need for programmes such as the English Cervical Screening Programme, to extend the screening interval for HPV negative women older than age 50 years to longer than the current five years.^23^ ^25^

In this pilot study, almost half of the women with HPV positive and cytology negative screening samples had a record of a negative HPV test by 24 months. Among those who attended their routine recall in three years (which took place four or five years after the HPV positive primary screen, depending on when the negative early recall test was recorded), 5.4 per 1000 women had CIN3+. Consistent with data from earlier small studies,^26^ ^27^ this incidence was about three times higher than after an HPV negative screen. The risk was in fact similar to that among 260 266 women younger than 50 years who had negative cytology and were screened again after a three year routine recall (4.5 with CIN3+ per 1000 screened in the second round, table 2). Hence, a routine recall interval not longer than three years would be appropriate for women in the English programme who are HPV negative after early recall.

APTIMA is currently the most widely used HPV mRNA test; in England, for example, the test is used for primary screening of more than half of all screened women.^28^ None of the randomised trials included APTIMA because the test was only made commercially available after 2010, when most trials had already concluded. This test’s sensitivity has been validated according to international non-inferiority validation guidelines, which require that newer HPV tests detect not only a similar number, but also the same CIN2+ cases as HPV tests validated in trials (Hybrid Capture 2 and GP5+/6+).^29^ A study in the Netherlands found that APTIMA’s relative sensitivity for CIN2+ was 0.97 compared with that of GP5+/6+, with 65 and 67 cases detected, respectively, out of 69 included in the study.^30^ Other previous longitudinal studies of APTIMA reported similar findings as our study, although those studies tended to be smaller and relied on the detection of cases defined with less stringent histological endpoints.^31^ ^32^ ^33^ ^34^ ^35^ Although our data stem from routine implementation where several factors could affect the detection of high grade lesions, our findings further strengthen this current evidence base. In the three to five years after a negative HPV test (depending on the woman’s age), neither the detection of CIN3+ at the second round nor the frequency of interval cancer incidence showed that the risk of a clinically relevant lesion was increased for APTIMA compared with the DNA tests. Consequently, these data do not suggest that a shorter routine recall interval should be used for women who test negative on APTIMA in comparison with women who test negative on cobas or RealTime HPV DNA tests.

Screening programmes are now including an increasing number of young women who had been vaccinated against HPV16/18 as adolescents. Vaccinated women have a substantially lower risk of CIN3+^36^; therefore, routine recall intervals longer than five years are highly likely to be safe for these women.^37^ However, HPV self-sampling is becoming an increasingly desirable alternative for taking a screening sample, for which most data for test accuracy rely on cross sectional detection after a positive test.^38^ The longitudinal performance after a negative test will need to be monitored for this sampling method.

### Conclusions

Using the data from the English HPV screening pilot, we conclude that the low risk at three years of CIN3+ and cervical cancer after a negative HPV test for women younger than 50 years compared with the risk after negative cytology test supports the extension of the three year screening interval to at least five years, which has been planned by the English Cervical Screening Programme. Although the difference in the risks between HPV testing and cytology was smaller in women aged 50 years and older, the risk at five years of CIN3+ after a negative HPV test was halved compared with the risk at three years in women younger than 50 years. This finding suggests that the current interval of five years at age 50 years and older could also be extended. The same data suggest that retaining a shorter recall interval of three years would be advisable for women with negative HPV tests at early recall and no cytological abnormalities. Our data also suggest that the same screening intervals can be used for women screened with APTIMA as are used for women screened with cobas or RealTime HPV DNA tests.

**What is already known on this topic**

Randomised trials including about 176 000 women have shown that human papillomavirus (HPV) testing is more sensitive than testing of cytology (known as smear tests) for the detection of high grade, cervical intraepithelial neoplasia
These trials showed that the risk of cervical cancer is lower for longer than three years after a negative HPV test result than after a negative cytological result, which supports the extension of HPV screening intervals
In England, Scotland, and Wales, HPV testing has now replaced cytological tests as the primary cervical screening test, and evidence based decisions regarding interval extension are therefore timely

**What this study adds**

This real life study provides evidence to support the extension of primary screening intervals for women who test negative on an HPV test at any age
For women who test negative 12 or 24 months after a positive HPV screening test, consideration by policy makers to retain current screening intervals might be warranted
These data are some of the strongest to suggest that primary screening intervals do not need to vary for DNA or mRNA HPV tests, provided that the tests are clinically validated by international metrics and are scalable for screening use

## Data Availability

The data belong to the former Public Health England and the authors cannot provide access to the relevant datasets to third parties. Requests for data and pre-application advice should instead be made to Office for Data Release (ODR@phe.gov.uk).

## Web extra

Extra material supplied by authors

Web appendix: Online appendix

## Notes

Contributors: CM and FP contributed equally. KD and HK contributed equally. The HPV pilot steering group designed the pilot study. MR undertook statistical analyses, wrote the original draft, and finalised the manuscript, was involved in the decision to submit, and is the guarantor. CM provided data management, reviewed the manuscript, and was involved in the decision to submit. FP helped with the statistical analyses, reviewed the manuscript, and was involved in the decision to submit. KC, KD, and HK reviewed the manuscript, and were involved in the decision to submit. The corresponding author attests that all listed authors meet authorship criteria and that no others meeting the criteria have been omitted.

The lead author (the manuscript’s guarantor) affirms that the manuscript is an honest, accurate, and transparent account of the study being reported; that no important aspects of the study have been omitted; and that any discrepancies from the study as originally planned (and, if relevant, registered) have been explained.

Dissemination to participants and related patient and public communities: The findings from the pilot study will continue to be disseminated at scientific conferences and other professional meetings. The dissemination to organisations and professional groups such as the English Cervical Screening Programme, UK National Screening Committee, British Society for Colposcopy and Clinical Pathology, and the British Association for Cytopathology have formed the basis for approval and implementation of changes in policy recommendations. The findings have been discussed with patient charities such as Jo’s Cervical Cancer Trust. As a leading cervical cancer charity supporting and advising women, Jo’s Cervical Cancer Trust have been active in supporting cervical screening programmes in Wales and Scotland in communication around interval extension. The findings will be, furthermore, publicised on social media belonging to the authors and/or their employers.

Provenance and peer review: Not commissioned; externally peer reviewed.
