# Supplementary material for: Extension of cervical screening intervals with primary human papillomavirus testing: observational study of English screening pilot data
Source: BMJ. 2022 May 31;377:e068776. doi: 10.1136/bmj-2021-068776 (PMC9153243; doi:10.1136/bmj-2021-068776)

London, 7 February 2022

EXTENSION OF CERVICAL SCREENING INTERVALS WITH PRIMARY HUMAN PAPILLOMAVIRUS TESTING: AN OBSERVATIONAL STUDY OF ENGLISH SCREENING PILOT DATA

SUPPLEMENTARY INFORMATION

Matejka Rebolj, Kate Cuschieri, Christopher S Mathews, Francesca Pesola,
Karin Denton, Henry Kitchener, on behalf of the HPV pilot steering group

The characteristics of the women included in the analyses

Table S1. Distribution of age and deciles of index of multiple deprivation, by screening round and screening test.

	**First round**		**Second round**	
	**HPV test**	**Cytology**	**HPV test**	**Cytology**
**Total**				
**Age group (years)**				
24-49	300,677 (74%)	706,820 (75%)	188,318 (88%)	260,266 (92%)
50-59	79,040 (20%)	175,973 (19%)	24,550 (12%)	22,195 (8%)
60-64	24,166 (6%)	54,908 (6%)	NR	NR
**IMD decile**				
1-5 (more deprived)	205,241 (51%)	555,030 (59%)	105,532 (50%)	165,039 (58%)
6-10 (less deprived)	198,642 (49%)	382,671 (41%)	107,336 (50%)	117,422 (42%)

Abbreviations. HPV: human papillomavirus. IMD: index of multiple deprivation. NR: not relevant (women were not eligible for a new invitation to primary screening).

The characteristics of the women attending the pilot's second screening round

Roughly similar proportions of women screened negative with HPV testing vs. cytology in the pilot's first round attended the second round (Table S2). Among women aged 24-49, 76% of those who were tested for HPV in the pilot's first screening round also attended screening in the pilot's second round. Among women screened with cytology, this was 70%. At age 50-59 years, the proportions were 34% for HPV testing and 30% for cytology. More women screened with cytology were excluded from the analysis of the second screening round, as their screening test was changed to HPV. However, women who were screened with the same test in the second round had a very similar distribution of the IMD as all women who attended the second round (Table S3). This was observed in both age groups and for both screening tests. As an example, among women aged 24-49 screened with HPV testing in the first round, 50% of those attending the second round with any screening test were categorised as "less deprived"; among the subgroup who were screened with HPV testing in both rounds, this was also 50%. Among women aged 24-49 screened with cytology in the first round, 42% of those attending the second round with any screening test were categorised as "less deprived"; among the subgroup who were screened with cytology in both rounds (representing 56% of women screened in the second round, see Table S2), this was 41%.

Table S2. The proportions of women with negative screening tests attending any screening in the pilot's second round, and the subgroup who continued to be screened with the same type of a screening test.

**Age group (years)**	**Were screened in the second round with any test**		**Were screened in the second round with the same test**	
	**HPV testing (%)[a]**	**Cytology (%)[a]**	**HPV testing (%)[b]**	**Cytology (%)[b]**
24-49	194,209 (76%)	467,972 (70%)	188,318 (97%)	260,266 (56%)
50-59	25,082 (34%)	52,006 (30%)	24,550 (98%)	22,195 (43%)

Abbreviations. HPV: human papillomavirus.
[a] Of women with negative screening tests in the pilot's first round (not tabulated).
[b] Of women screened in the second round.

Table S3. IMD distribution among those who attended primary screening in the pilot's second screening round, by age group and screening test.

Type of screening test	Age group (years)	Screening test in the first round	IMD dummy	N attending in the second round	% of N attending in the second round	Screening test in the first round	IMD dummy	N attending in the second round	% of N attending in the second round
**Any**	**24-49**	**HPV**	**More deprived**	97,892		**Cytology**	**More deprived**	273,297	
			**Less deprived**	96,317	50%		**Less deprived**	194,675	42%
**Same as the first round**	**24-49**	**HPV**	**More deprived**	94,900		**Cytology**	**More deprived**	153,342	
			**Less deprived**	93,418	50%		**Less deprived**	106,924	41%
**Any**	**50-59**	**HPV**	**More deprived**	10,889		**Cytology**	**More deprived**	26,835	
			**Less deprived**	14,193	57%		**Less deprived**	25,171	48%
**Same as the first round**	**50-59**	**HPV**	**More deprived**	10,632		**Cytology**	**More deprived**	11,697	
			**Less deprived**	13,918	57%		**Less deprived**	10,498	47%

Note. IMD dummy: "more deprived" includes IMD deciles 1-5, whereas "less deprived" includes IMD deciles 6-10.
Example. Among women who were aged 24-49 years when they were screened with HPV testing in the pilot's first round, 194,209 (97,892+96,317) attended the second screening round. Among the latter, 96,317 (50%) were from the less deprived areas. Of the 194,209 women, 188,318 (94,900+93,418, or 97%, see also Table S2) were again screened with an HPV test. Among these 188,318 women, 93,418 (50%) were from the less deprived areas.

Table S4. Unadjusted OR and HR from Tables 2 and 4 in the main text, comparing the detection of CIN3+ and cervical cancer after primary screening with HPV testing with primary screening with cytology, by age group.

	**24-49 years**		**50-59 years**		**60-64 years**	
	**Unadjusted estimate (95% CI)**	**Adjusted estimate (95% CI)**	**Unadjusted estimate (95% CI)**	**Adjusted estimate (95% CI)**	**Unadjusted estimate (95% CI)**	**Adjusted estimate (95% CI)**
**First round**						
After a non-negative screen						
CIN3+ (OR)	1.53 (1.47-1.58)	1.55 (1.50-1.61)	1.47 (1.24-1.74)	1.56 (1.31-1.85)	1.70 (1.22-2.36)	1.69 (1.21-2.37)
Cervical cancer (OR)	1.38 (1.18-1.61)	1.38 (1.18-1.61)	1.33 (0.85-2.07)	1.41 (0.90-2.21)	0.89 (0.41-1.92)	--
After a negative screen[b]						
Cervical cancer (HR)	0.45 (0.23-0.85)	0.44 (0.23-0.84)	0.26 (0.06-1.15)	--	0.39 (0.05-3.20)	--
**Second round**[c]						
After a non-negative screen						
CIN3+ (OR)	0.27 (0.23-0.31)	0.26 (0.23-0.30)	0.63 (0.32-1.25)	--	NR	NR
Cervical cancer (OR)	0.03 (0.00-0.18)	0.02 (0.00-0.17)	0.45 (0.04-4.98)	--	NR	NR

Abbreviations. CI: confidence interval. CIN: cervical intraepithelial neoplasia. HPV: high-risk human papillomavirus. HR: hazard ratio. NR: not relevant (women were not invited for the second round due to their age). OR: odds ratio.
Note. Estimates in red were reported in Tables 2 and 4. “--” means that the observed numbers of cases did not suffice for regression models with adjustments.

Table S5. Unadjusted OR and HR from Table 3 in the main text, comparing the detection of CIN3+ and cervical cancer with specific HPV assays, for women aged 24-59 years.

	**APTIMA vs. cobas**		**RealTime vs. cobas**		**APTIMA vs. RealTime**		**APTIMA vs. RealTime and cobas combined**	
	**Unadjusted estimate (95% CI)**	**Adjusted estimate (95% CI)**	**Unadjusted estimate (95% CI)**	**Adjusted estimate (95% CI)**	**Unadjusted estimate (95% CI)**	**Adjusted estimate (95% CI)**	**Unadjusted estimate (95% CI)**	**Adjusted estimate (95% CI)**
**First round**								
After a non-negative screen								
CIN3+ (OR)	1.06 (0.98-1.15)	1.03 (0.95-1.12)	0.92 (0.86-0.98)	1.06 (0.99-1.13)	1.15 (1.06-1.25)	1.00 (0.92-1.09)	1.11 (1.03-1.19)	1.04 (0.96-1.12)
Cervical cancer (OR)	1.14 (0.78-1.68)	1.15 (0.78-1.70)	1.33 (1.01-1.75)	1.44 (1.10-1.90)	0.86 (0.60-1.24)	0.80 (0.60-1.24)	0.97 (0.69-1.38)	0.95 (0.67-1.34)
After a negative screen[b]								
Cervical cancer (HR)	0.35 (0.04-2.84)	--	0.40 (0.12-1.33)	--	0.73 (0.08-6.21)	--	0.51 (0.07-3.91)	--
**Second round**[c]								
After a non-negative screen								
CIN3+ (OR)	1.46 (0.97-2.21)	1.38 (0.91-2.08)	1.47 (1.09-1.98)	1.65 (1.22-2.23)	1.00 (0.68-1.45)	0.86 (0.59-1.25)	1.16 (0.81-1.66)	1.05 (0.73-1.50)
Cervical cancer (OR)	--	--	0.78 (0.05-12.41)	--	--	--	--	--

Abbreviations. CI: confidence interval. CIN: cervical intraepithelial neoplasia. HPV: high-risk human papillomavirus. HR: hazard ratio. OR: odds ratio.
Note: estimates in red were reported in Table 3. "--" means that the observed numbers of cases did not suffice for regression models (with or without adjustments, as relevant).

Figure S1. Completeness of data in the study, by age group and calendar year of the primary screening test in the first round.

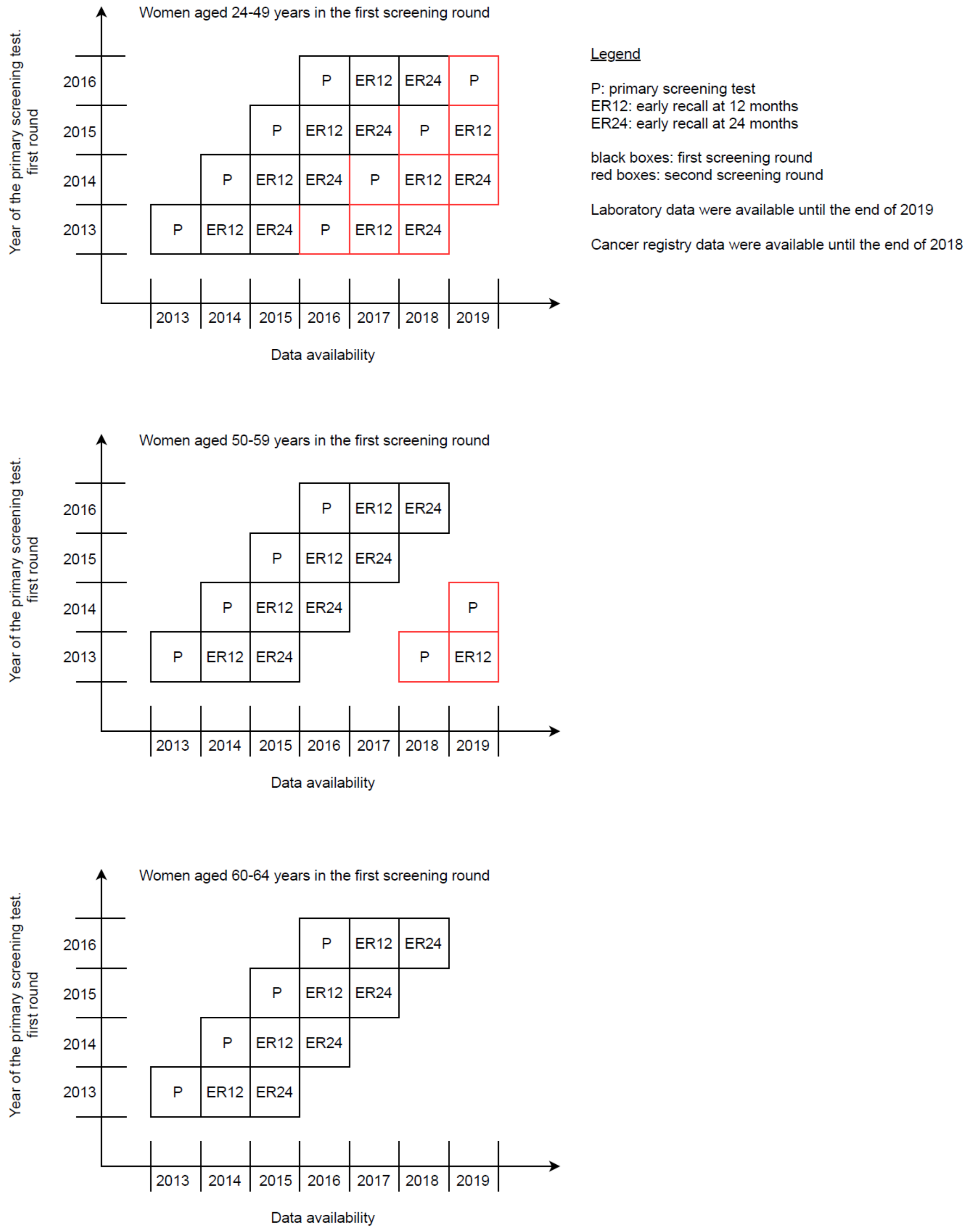

Figure S2. Flow chart.

Note that the flowchart does not include women who were not included in the analyses e.g., those with inadequate test results or those who defaulted from the recommended follow-up with early recall or colposcopy.

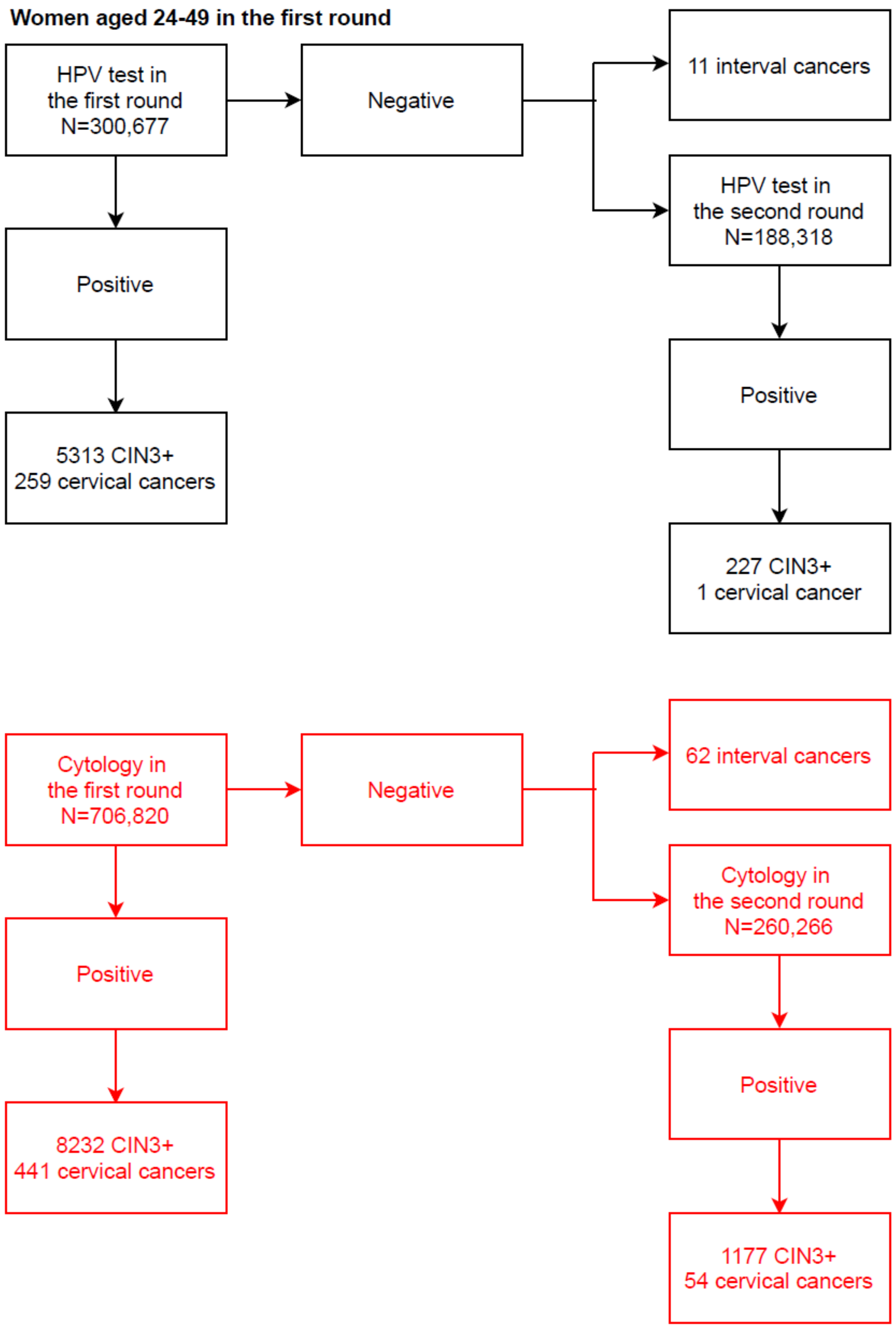

Women aged 50-59 in the first round

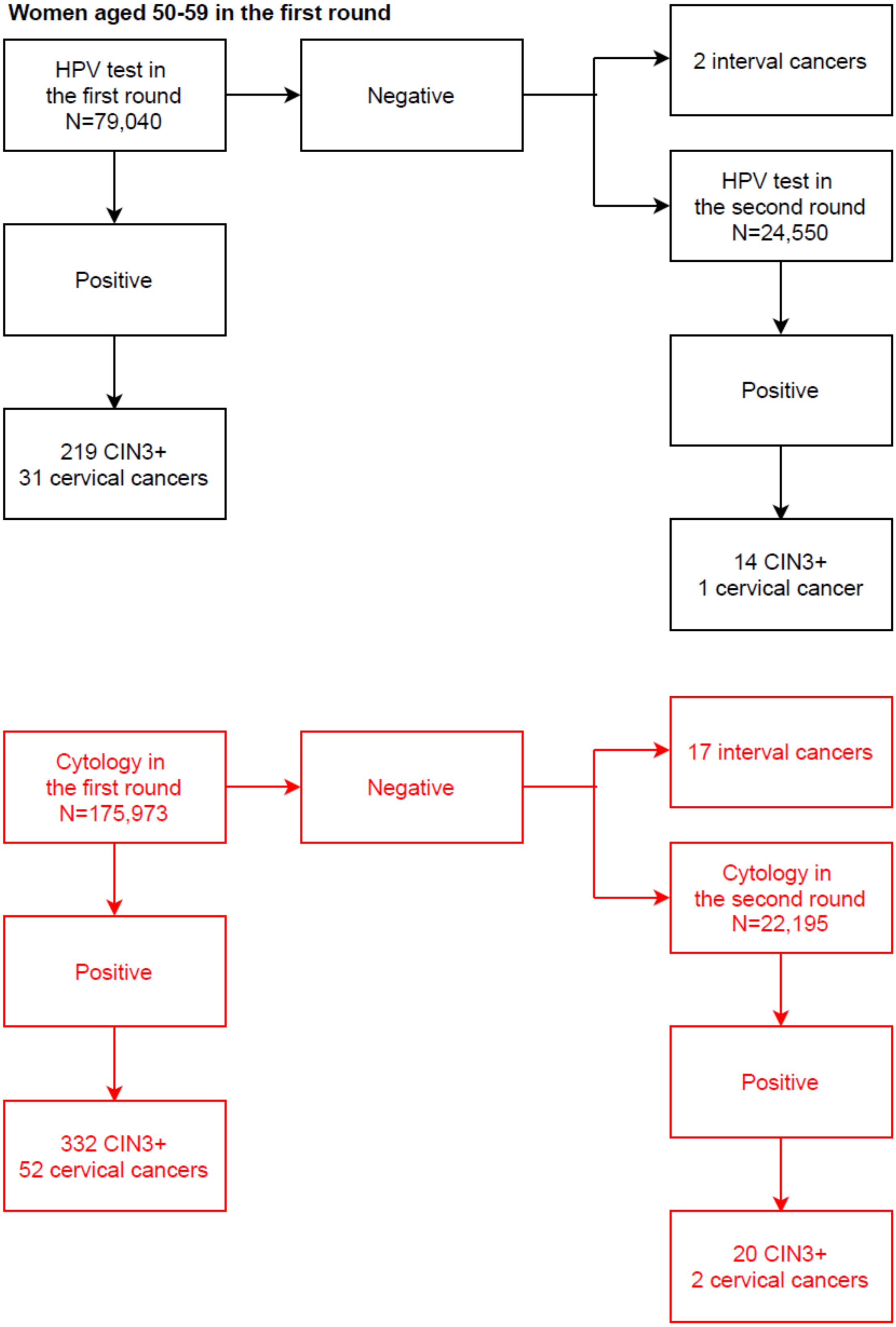

Women aged 60-64 in the first round

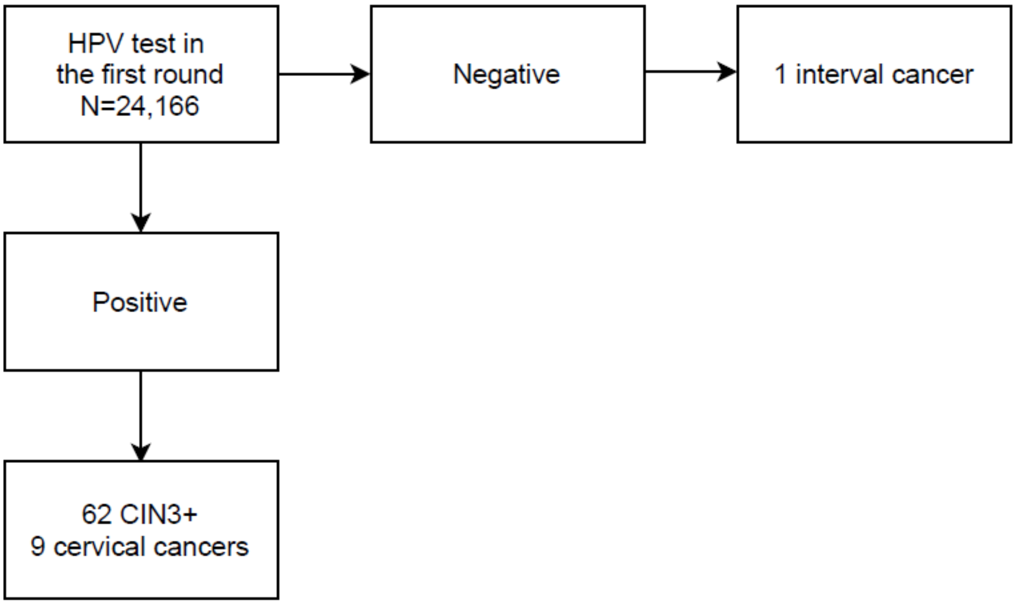

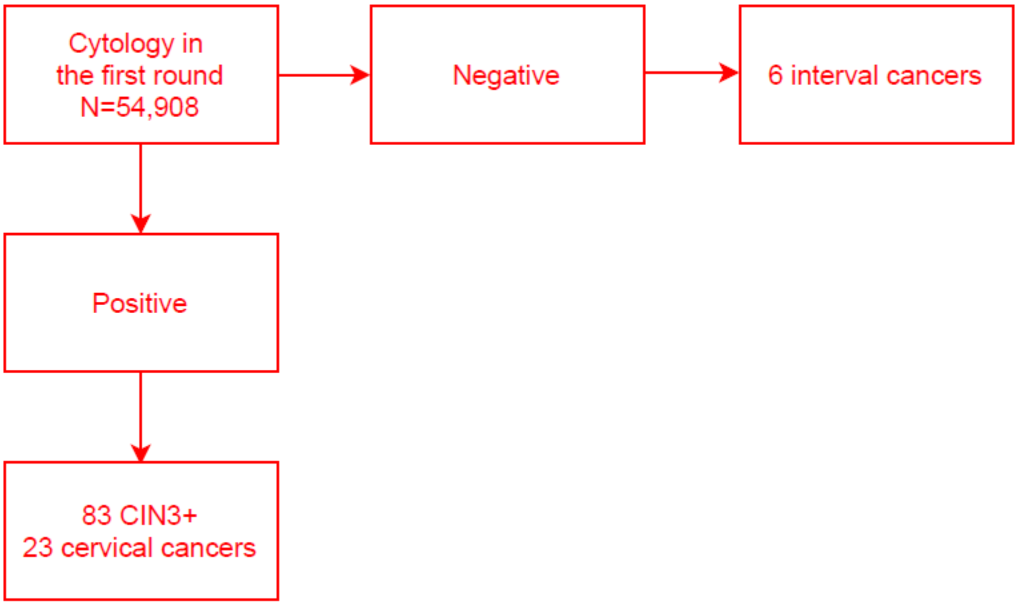

Supplement: Supplementary file 1 — Web appendix: Online appendix [file rebm068776.ww.pdf]
